# Meeting the WHO 24-h guidelines among 2–6-year-old children by family socioeconomic status before and during the COVID-19 pandemic: a repeated cross-sectional study

**DOI:** 10.1186/s44167-022-00010-4

**Published:** 2023-01-03

**Authors:** Henriikka Koivukoski, Elina Hasanen, Asko Tolvanen, Terence Chua, Michael Chia, Hanna Vehmas, Arja Sääkslahti

**Affiliations:** 1 Faculty of Sport and Health Sciences, University of Jyväskylä, Jyväskylä, Finland; 2 Faculty of Education and Psychology, University of Jyväskylä, Jyväskylä, Finland; 3 National Institute of Education, Nanyang Technological University, Singapore, Singapore

**Keywords:** Lifestyle habits, 24-h activity guidelines, Screen time, Physical activity, Sleep, Socioeconomic status, Household income, Parent education, Young children, COVID-19

## Abstract

**Background:**

The World Health Organization (WHO) has developed guidelines for 24-h physical activity (PA), sedentary behaviour and sleep for young children. Lower socioeconomic status (SES) has been linked to a lower likelihood of meeting these guidelines. The outbreak of the novel coronavirus disease (COVID-19) raised concerns about young children’s opportunities to meet the guidelines. The study focused on the prevalence of meeting the WHO’s 24-h guidelines on screen time (ST), PA and sleep among 2–6-year-old children, in association with family SES, before COVID-19 outbreak in 2019, and during the pandemic in 2020 and 2021 in Finland.

**Methods:**

Data were collected at three timepoints by an online survey through day-care centres. Meeting the WHO 24-h guidelines was defined for each behaviour, from a parent-reported seven-day recall of a typical day on weekdays and weekend days and adapted to the national context. Children were considered to meet the ST guideline if they had maximum of 60 min of ST, the PA guideline if they had minimum of 60 min of outdoor PA, and the sleep guidelines if they had minimum of 11/10/9 h (2/3–5/6 years) of good or very good quality sleep. Binary logistic regression models were used to examine the odds ratios of meeting the guidelines.

**Results:**

The prevalence of meeting the ST guideline was highest before the COVID-19 pandemic in 2019. The PA guideline was most met during the strict pandemic restrictions in 2020. Children from higher SES families were more likely to meet the ST and sleep duration guidelines either on weekdays or weekends. The PA guideline was met more on weekdays by children whose parents had lower education levels at all timepoints. In 2020, sleep quality guideline was less likely met by children with parents with the highest education levels.

**Conclusion:**

Higher SES may increase the odds of young children meeting the ST and sleep duration guidelines, but the results are more complex regarding PA and SES. The impact of the COVID-19 pandemic on ST, outdoor PA, and sleep of young children varied by family SES, and further research is recommended to identify causality of these relationships.

**Supplementary Information:**

The online version contains supplementary material available at 10.1186/s44167-022-00010-4.

## Background

Screen time (ST), physical activity (PA) and sleep are invariably intertwined in early childhood and are associated directly and indirectly with various health outcomes [1–8]. Screen time—referring to screen viewing or digital media use for various purposes—makes up a significant portion of children’s sedentary behaviour during their waking hours [1] and is linked, for example, to poorer sleep [2–4] which in turn, is connected, for instance, to unfavourable body composition and emotional regulation [5]. Individual health benefits of high PA [6, 7] and low sedentary time [8] are shown already at an early age. The significance of ST, PA and sleep for young children’s health is widely recognized; therefore, the World Health Organization (WHO) [9] has developed guidelines for 24-h physical activity, sedentary behaviour and sleep for children under five. Guidelines for children from 5–6 years exist in the WHO guidelines on physical activity and sedentary behaviour [10] and the Finnish national recommendations for physical activity in early childhood [11]. These sedentary behaviour guidelines are directed towards sedentary and recreational ST. Meeting the WHO’s 24-h guidelines regarding ST, PA and sleep have been found to enhance favourable outcomes in cardiometabolic health [12–14], motor development [15] and psycho-social health in young children [16–18].

### WHO 24-h guidelines for screen time, physical activity and sleep

For children under five years, the daily guidelines recommend no more than 60 min of sedentary ST and at least 180 min of PA in a variety of types spread throughout the day, with children aged 3–4 years spending at least 60 min in moderate-to-vigorous–intensity PA [9]. Good-quality sleep should cover 11–14 h a day for two-year-olds (including naps) and 10–13 h for 3–4-year-olds (with or without naps), with regular sleep and wake-up times. For children aged five years and above, the guidelines are to have at least 60 min of moderate-to-vigorous PA per day and to limit ST, especially recreational ST, but there is no specific maximum set for this age group [10]. National guidelines, however, recommend limitation of continuous sedentary time, such as using digital media, to 1 h for children under seven [11]. Regarding sleep, there is evidence that 5-year-olds need at least 10 h and 6-year-olds need 9 h of good-quality sleep per day [19].

### Adherence to the WHO lifestyle guidelines

The prevalence of meeting the WHO guidelines for ST, PA and sleep varies according to the measurement method and age groups of participants in the research. As a computed average from 11 different countries, 13 percent of under 5-year-old children meet all the guidelines [20], when, for example, in some urban areas outside of Europe rates are reported to be as low as three percent [21, 22]. Compared with PA and sleep, the ST guideline is met more rarely [12, 20, 22, 23]. In Finland, the prevalence of meeting all the guidelines is reduced from 52 percent at the age of 6 years to 25 percent at the age of 8 years [13]. This is in agreement with research showing that ST in children increases with age [24, 25], a key behavioural component of the WHO 24-h guidelines. Apparently, the prevalence of children under six meeting the WHO 24-h guidelines in Finland is not yet publicized, so research attention in this area is welcome.

### Socioeconomic differences in screen time, physical activity and sleep in young children

The socioeconomic status (SES) of the family may play an important role in young children’s ST, PA and sleep, and the situation among young children has not yet been established in research. A high household income level is associated with better adherence and a low income level with poorer adherence to the guidelines among children and youth aged from 5 to 17 years [26]. Higher parental education levels are found to diminish ST in toddlers of 3 years or under [27, 28] and 3–6-year-old children [29]. However, no parental education–level differences are found in children’s PA or other sedentary time [29]. Children aged 3–6 years and living in high-income settings are reported to exceed ST guidelines and still meet sleep guidelines more often than their counterparts living in low-income settings [30]. Elsewhere, higher income levels are favourably associated with a child’s ST [2] and lower sedentary behaviour but also with higher sleep variability in children aged 3–5 years [31]. Poverty is reported to be associated with low levels of moderate to vigorous PA in 1–3-year-old toddlers [32]. Lower parental education and later bedtimes are associated with high ST in 5-year-old children [33]. In later childhood and adolescence, low parental SES has been reported to be associated with low PA and high ST [1, 26, 34].

### Impact of COVID-19 situation on young children’s screen time, physical activity and sleep

The outbreak of coronavirus disease (COVID-19) has raised concerns about young children’s ability to meet the guidelines, particularly during the behavioural restrictions imposed [35, 36]. There are reports of 3–6-year-old children’s PA decreasing during COVID-19 and ST or sedentary behaviour increasing [37–40], as well as later bedtimes [38] and lower sleep efficiency [39]. However, there are also reports of children spending more time sleeping and in moderate-to-vigorous PA and less time in sedentary behaviour during the pandemic [40]. During the COVID-19 pandemic, for example in Spain, 0–12-year-old children’s sleep time was negatively correlated to their ST [41]. Furthermore, children who were able to spend time outdoors were more likely to meet all three guidelines than children whose freedom of movement was more restricted [42].

## Materials and methods

The purpose of this study was to examine (1) the prevalence of 2–6-year-old Finnish children meeting the WHO 24-h guidelines for ST, PA and sleep and (2) the association of family SES with meeting the WHO guidelines. The study took place in 2019, 2020 and 2021, including a period of relatively strict restrictions in November 2020, when there were strong advisories for social distancing (including restrictions on organized PA), remote work and self-quarantining at home, with some day-care centre groups quarantined due to exposure to the virus. There was great areal variability in the epidemic situation around the country and in the restrictions imposed, particularly during the third data collection in November–December 2021, when the pandemic had continued for over a year. Overall, in Finland, the spread of COVID-19 followed the progression of the pandemic in other EU countries but with a lower incidence of infections; therefore, no complete lockdowns were imposed [43].

### Sampling procedures

We gathered randomly selected, nationally representative, cross-sectional data of parents and guardians of children attending early childhood education, to investigate children’s ST, outdoor PA and sleep at three timepoints. The data collection lasted three weeks each year and was timed for November–December. The data were collected from 59 municipalities (out of Finland’s 310 municipalities) through day-care centres and pre-schools from parents of 2–6-year-old children using an online survey platform (Qualtrics). The municipalities were randomized by the probability proportional to size (PPS) sampling method and, after obtaining research permission from each, were included in the study. A total of two dropouts were replaced by a municipality from the same geographical area. The sampling procedures have been reported elsewhere [44].

### Ethics

The research complied with the ethical principles outlined in the Declaration of Helsinki and received institutional ethical approval (NTU IRB 2019-02-036). The parents received an invitation to participate in the study through their early childhood education online communication systems and, in one municipality, on paper. The parents were informed that participation was voluntary and anonymous. The voluntariness of the participation was confirmed by parental consent before they completed the survey.

### Survey Instrument

Surveillance of digital Media in eArLy ChiLdhood Questionnaire (SMALLQ®) was developed to solicit information from parents about the digital (ST, type and purpose) and non-digital media (indoor and outdoor play, sleep duration and quality) habits of their children on a weekday and on the weekend, their attitudes and concerns about child digital media use, and their awareness and practice of child media use guidelines [45]. It was originally developed and validated in Singapore and the development and validity process is previously described by Chia, Tay and Chua [45].

Face and content validity of the questionnaire items in SMALLQ® were established by a number of independent experts based upon the criteria of representativeness, clarity, and relevance [46] as well as by feedback solicited from a group of parents of preschool children who were not involved in the research. The internal consistency of Finnish data was computed as 0.72, based upon self-reported parent and child digital media use on the weekday and weekend. Cronbach’s alpha values of 0.70 to 0.90 are deemed to be valued and acceptable [51]. In summary, the online SMALLQ® was adjudged to have sufficient face and content-validity with acceptable internal consistency or reliability.

The original language of SMALLQ® was Singaporean English. For this study, it was translated to the native languages of Finland (Finnish and Swedish) and minority language (Russian) by a panel of local experts using the established WHO method for cultural adaptation [47]. SMALLQ® was then sent out to the target population in four different languages including English.

### Description of the variables

The outcome variables analyzed in this research were the child’s digital media use in total (considered here as ST), outdoor physical play, sleep duration and sleep quality. Outdoor PA has been shown to indicate total PA [48–50], and outdoor play is emphasized in the national recommendations of PA for under school-aged children [11]. Regarding the child’s digital media use, parents were asked to estimate their child’s digital media use outside of pre-school/day-care, for example, at home. They filled in the hours and minutes, after the statement “In the last 7 days, my child spent time in…” for a typical weekday and a typical weekend day, in five different digital media activities: (1) using media for education/learning, (2) using media for entertainment, (3) creating media, (4) communicating and (5) others. The child’s outdoor physical play was estimated (alongside six other non-digital media activities) in a similar way by a parent’s recall of the last 7 days, apart from pre-school/day-care, as were night- and daytime sleep durations. The overall quality of the child’s night-time sleep was scaled in five levels: (1) very poor, (2) quite poor, (3) average, (4) good and (5) very good.

Explanatory variables were the age and gender of the child and parent and SES, determined by parental education and household income levels. In the questionnaire, the options for parental education levels were: (1) No formal education, (2) Primary/elementary school, (3) Secondary/high school, (4) Vocational school, (5) Bachelor’s degree and (6) Master’s degree or higher. Household’s income level was formed according to Finnish income levels [51]. For the analysis, levels 1–3 of parental education and levels 1–2 (two lowest-income levels) and 6–8 (three highest-income levels) of household income were combined, respectively.

### Benchmarking against the WHO 24-h guidelines

ST, outdoor PA, sleep duration and sleep quality were recoded as binary variables, with values of 0 = the guidelines are not met and 1 = the guidelines are met. Child’s ST was calculated as a sum of all different digital media activities, separately for a typical weekday and a typical weekend day. The limit for meeting the ST guideline was set to ≤ 1 h, according to the WHO 24-h guidelines and the Finnish national recommendations [11]. For the PA guideline, the limit was set to ≥ 1 h of outdoor PA, adapted from the WHO 24-h guidelines, acknowledging the average time children spend on PA during pre-school/day-care [52] and recognizing that Finnish children’s outdoor play is mostly moderate-to-vigorous PA [53]. The limit of meeting the sleep duration guideline was set to ≥ 11 h for 2–year-olds, ≥ 10 h for 3–5-year-olds, and ≥ 9 h for 6-year-olds, adapted from the WHO 24-h guidelines and Galland et al. [19]. The limit for meeting the sleep quality guideline was set to 4) good or 5) very good, according to the WHO 24-h guidelines.

### Statistical analyses

For the statistical analyses, Mplus was used [54]. We used binary logistic regression models to find odds ratios in meeting the guidelines, together with estimating the full information maximum likelihood. Explanatory variables (parental education, household income, parental age and gender, child’s age and gender) were put in the model simultaneously (Additional file 1). To make sure that the missing at random (MAR) assumption is met, the parental concern variable should be added to the model as an auxiliary variable [55, 56]. When missing data of outcome variables and other variables of the dataset were analysed, a variable of parental concerns on their child’s digital media use was a statistically significant predictor of missing values (Additional file 1). Statistical significance was set at p < 0.05.

## Results

The sample size was 2529 in 2019, 5472 in 2020 and 5103 in 2021 (13,104 in total). Of the parents, 84 percent were mothers, and of the children, 52 percent were boys (Table 1). Guardians other than mothers and fathers and child’s genders other than girls and boys were excluded from the analysis due to their low frequency. The mean age of the parents was 36.5 (5.64 SD), and that of the children was 4.4 (1.33 SD). A bachelor’s degree was held by 37 percent of parents. Table 1 shows the demographics of the participants in the study.

**Table 1:** Demographics of the parents and their children in the study from 2019–2021

	2019 N (%)	2020 N (%)	2021 N (%)	All N (%)
Child’s information				
Boy	933 (51.6)	1935 (50.9)	1846 (51.0)	4714 (51.5)
Girl	874 (48.3)	1858 (48.9)	1767 (48.8)	4499 (48.8)
Other	2 (0.1)	5 (0.1)	6 (0.2)	13 (0.1)
Mean age (SD)	4.5 (1.23)	4.4 (1.36)	4.4 (1.33)	4.43 (1.33)
Parent’s information				
Mother	1507 (83.7)	3233 (89.1)	2971 (82.4)	7711 (84)
Father	282 (15.7)	527 (9.6)	607 (16.8)	1416 (15.4)
Other	11 (0.6)	18 (0.3)	26 (0.7)	55 (0.6)
Mean age (SD)	36.2 (5.42)	36.1 (5.55)	37.1 (5.81)	36.5 (5.64)
Parental education level				
1) No formal education or Primary/Elementary school	46 (2.6)	76 (2.0)	65 (1.8)	187 (2.0)
2) Secondary/High school	91 (5.1)	180 (4.8)	188 (5.2)	459 (5.0)
3) Vocational school	437 (24.3)	856 (22.7)	719 (20.0)	2012 (22.0)
4) Bachelor’s degree	669 (37.2)	1429 (37.9)	1273 (35.4)	3371 (36.8)
5) Master’s degree	553 (30.8)	1230 (32.6)	1351 (37.6)	3134 (34.2)
Household income level (€/year)				
1) 0–19,999	143 (8.1)	305 (8.2)	242 (6.8)	690 (7.6)
2) 20,000–39,999	273 (15.4)	523 (14.1)	497 (14.0)	1293 (14.3)
3) 40,000–69,999	598 (33.8)	1262 (34.0)	1043 (29.5)	2903 (32.2)
4) 70,000–99,999	490 (27.7)	1049 (28.2)	1001 (28.3)	2540 (28.1)
5) 100,000–119,999	129 (7.3)	280 (7.5)	353 (10.0)	762 (8.4)
6) 120,000 or more	138 (7.8)	298 (8.0)	405 (11.4)	841 (9.3)

Table 2 shows that the percentage of meeting the ST guideline was higher on weekdays than on weekends in all timepoints. On weekdays, the adherence was higher in 2019 (42%) and 2021 (40%) than in 2020 (36%). On weekends, the adherence was highest in 2019 (21%), while only 17% met the guideline in 2020 and 2021. The PA guideline was met more often in 2020 (62%) compared with the other two years (56%) on weekdays, and for weekends, in 2020 (92%) and 2021 (89%) compared to 2019 (85%). The guideline for sleep duration was met, with nearly four-fifths of the total sample attaining them on weekdays and with 90 percent of the total sample on weekends. Sleep quality was good or excellent for 82–85 percent of the children on weekdays and for 87–90 percent on weekends. Meeting the sleep quality guideline was nearly five percentage points lower in 2021 compared with the previous two years.

**Table 2:** The percentages of young children meeting the WHO 24-h guidelines in 2019–2021 on a typical weekday and a typical weekend day

	2019 % (N)	2020 % (N)	2021 % (N)	Wald X^2^	p-value	Pairwise comparison
ST^a^						
Weekday	42.3 (746)	36.1 (1365)	39.9 (1436)	23.07	< 0.001*	2020 < 2019, 2021
Weekend	20.6 (370)	16.6 (626)	16.6 (595)	14.16	< 0.001*	2019 > 2020, 2021
Outdoor PA^b^						
Weekday	55.9 (991)	61.8 (2313)	55.9 (1967)	36.02	< 0.001*	2020 > 2019, 2021
Weekend	85.3 (1530)	91.8 (3465)	89.4 (3195)	53.59	< 0.001*	2019 < 2020, 2021
Sleep duration^c^						
Weekday	80.1 (1446)	79.1 (2997)	78.5 (2835)	1.69	.429	
Weekend	90.2 (1628)	90.1 (3411)	89.7 (3230)	0.43	.804	
Sleep Quality^d^						
Weekday	85.1 (1534)	85.0 (3219)	81.8 (2947)	17.27	< 0.001*	2019 < 2021
Weekend	90.5 (1626)	89.6 (3383)	86.9 (3123)	20.37	< 0.001*	2019, 2020 > 2021

^a^ST: maximum of 60 min of total ST

^b^Outdoor PA: minimum of 60 min of outdoor PA

^c^Sleep Duration: minimum of 11 h of total sleep for 2-year-olds, 10 h for 3–5-year-olds, and 9 h for 6-year-olds

^d^Sleep Quality: minimum of good quality sleep; *Wald test is statistically significant at p < 0,05

Compared with mid-income households, children from higher-income households were 1.3 times more likely to meet the ST guideline on weekends in 2019 and weekdays in 2020 and 2021 (Table 3). Parents with a master’s degree or higher had their children 1.2–1.5 times more likely to meet the ST guideline than children of parents with a bachelor’s degree in 2021 and on weekdays of 2020.

**Table 3:** Coefficients and odds ratios (OR) of having maximum of 60 min of ST

	2019			2020			2021		
	Estimate	OR	95% CI	Estimate	OR	95% CI	Estimate	OR	95% CI
*Household income level (€/year)*									
1) Less than 20,000									
Weekday	− 0.083	0.920	0.611–1.385	− 0.134	0.874	0.655–1.166	− 0.010	0.990	0.719–1.363
Weekend	0.276	1.318	0.810–2.143	0.008	1.008	0.702–1.448	0.059	1.061	0.709–1.587
2) 20,000–39,999									
Weekday	− 0.080	0.923	0.684–1.246	− 0.153	0.858	0.683–1.079	0.105	1.111	0.880–1.403
Weekend	0.240	1.271	0.879–1.838	− 0.032	0.968	0.714–1.313	0.001	1.001	0.732–1.370
4) 70,000–99,999									
Weekday	0.065	1.067	0.825–1.380	0.159	1.173	0.981–1.402	0.142	1.153	0.955–1.393
Weekend	0.435*	1.545	1.121–2.130	0.120	1.127	0.896–1.418	− 0.067	0.935	0.724–1.209
5) 100,000 or more									
Weekday	0.067	1.070	0.778–1.470	0.270*	1.310	1.051–1.634	0.311*	1.364	1.106–1.683
Weekend	0.222	1.248	0.825–1.888	0.071	1.074	0.805–1.431	0.123	1.131	0.855–1.495
*Parental education level*									
2) No formal/Primary/Secondary									
Weekday	− 0.256	0.774	0.514–1.165	− 0.200	0.819	− 1.286–0.199	− 0.167	0.846	0.619–1.157
Weekend	0.046	1.047	0.635–1.728	− 0.042	0.959	0.642–1.431	− 0.066	0.936	0.629–1.394
3) Vocational									
Weekday	− 0.004	0.996	0.766–1.295	− 0.167	0.846	− 1.649–0.099	− 0.199	0.819	0.664–1.011
Weekend	0.066	1.068	0.775–1.472	− 0.048	0.953	0.733–1.240	− 0.177	0.838	0.630–1.115
5) Master’s degree or higher									
Weekday	0.199	1.220	0.955–1.559	0.181*	1.199	2.099–0.036	0.420**	1.522	1.286–1.803
Weekend	− 0.023	0.978	0.717–1.332	0.137	1.147	0.923–1.426	0.263*	1.301	1.037–1.633

Reference categories: Child’s gender: boy; Household income level: 3) 40,000–69,999 (€/year); Parental education level: 4) Bachelor’s degree. *p-value < 0.05, **p-value < 0.001

Compared with the mid-income households, children from higher-income households were less likely to meet the PA guideline on weekdays in 2020 and 2021, while children from lower-income levels had lower likelihood on the weekends of 2019 and 2021 (Table 4). On the weekends of 2020, children from the highest-income level households were 1.6 times more likely to spend at least 1 h on outdoor PA. Compared with parents with a bachelor’s degree, parents with vocational education levels increased the likelihood of children meeting the PA guideline on weekdays, and a master’s degree or higher decreased the likelihood. On the weekends of 2020, the lowest education level was connected to a 0.6 times lower likelihood of meeting the PA guideline.

**Table 4:** Coefficients and odds ratios (OR) of having minimum of 60 min of outdoor PA

	2019			2020			2021		
	Estimate	OR	95% CI	Estimate	OR	95% CI	Estimate	OR	95% CI
*Household income level (€/year)*									
1) Less than 20,000									
Weekday	− 0.256	0.775	0.523–1.147	0.017	1.018	0.767–1.350	0.173	1.188	0.868–1.627
Weekend	− 0.421	0.657	0.394–1.094	− 0.140	0.869	0.554–1.363	− 0.432	0.649	0.416–1.013
2) 20,000–39,999									
Weekday	− 0.047	0.954	0.705–1.291	− 0.136	0.873	0.700–1.088	− 0.052	0.949	0.756–1.191
Weekend	− 0.518*	0.596	0.403–0.880	− 0.103	0.902	0.624–1.304	− 0.481*	0.618	0.442–0.864
4) 70,000–99,999									
Weekday	0.103	1.108	0.858–1.431	− 0.200*	0.819	0.688–0.974	− 0.137	0.872	0.727–1.047
Weekend	− 0.123	0.885	0.613–1.276	− 0.081	0.923	0.680–1.252	− 0.174	0.840	0.621–1.137
5) 100,000 or more									
Weekday	− 0.110	0.896	0.653–1.230	− 0.161	0.851	0.686–1.055	− 0.218*	0.804	0.655–0.987
Weekend	− 0.002	0.998	0.637–1.563	0.463*	1.589	1.020–2.476	− 0.177	0.838	0.594–1.182
*Parental education level*									
2) No formal/Primary/Secondary									
Weekday	0.197	1.218	0.818–1.814	− 0.182	0.834	0.624–1.113	0.014	1.015	0.757–1.359
Weekend	0.114	1.121	0.653–1.923	− 0.588*	0.556	0.356–0.867	0.176	1.193	0.741–1.920
3) Vocational									
Weekday	0.467*	1.596	1.225–2.078	0.367**	1.443	1.187–1.754	0.358*	1.430	1.168–1.751
Weekend	0.213	1.237	0.853–1.795	− 0.192	0.825	0.590–1.154	− 0.109	0.896	0.659–1.220
5) Master’s degree or higher									
Weekday	− 0.270*	0.764	0.599–0.973	− 0.230*	0.794	0.674–0.936	− 0.237*	0.789	0.669–0.931
Weekend	− 0.114	0.892	0.643–1.238	− 0.119	0.887	0.654–1.204	0.011	1.012	0.776–1.319

Reference categories: Child’s gender: boy; Household income level: 3) 40,000–69,999 (€/year); Parental education level: 4) Bachelor’s degree. *p-value < 0.05, **p-value < 0.001

Children from higher-income households were more likely to meet the sleep duration guidelines than mid-income households on weekends (Table 5). On weekdays in 2021, children from lower-income households were less likely to meet the sleep duration guideline than mid-income households. In 2020, parents with lower education levels had their children less likely to meet the sleep duration guideline than parents with bachelor’s degrees.

**Table 5:** Coefficients and odds ratios (OR) of having a total sleep duration of 11/10/9 h (2/3–5/6 years)

	2019			2020			2021		
	Estimate	OR	95% CI	Estimate	OR	95% CI	Estimate	OR	95% CI
*Household income level (€/year)*									
1) Less than 20,000									
Weekday	− 0.278	0.757	0.472–1.215	0.133	1.142	0.822–1.587	− 0.377*	0.686	0.485–0.969
Weekend	− 0.537	0.585	0.338–1.010	0.060	1.062	0.698–1.615	− 0.092	0.912	0.579–1.437
2) 20,000–39,999									
Weekday	− 0.268	0.765	0.529–1.105	− 0.147	0.863	0.670–1.111	− 0.152	0.859	0.657–1.122
Weekend	0.382	1.465	0.857–2.503	− 0.067	0.935	0.676–1.293	0.041	1.042	0.736–1.474
4) 70,000–99,999									
Weekday	− 0.011	0.989	0.719–1.360	0.117	1.125	0.911–1.389	0.108	1.114	0.891–1.391
Weekend	0.235	1.265	0.828–1.935	0.310*	1.364	1.018–1.827	0.475*	1.608	1.182–2.186
5) 100,000 or more									
Weekday	0.285	1.329	0.858–2.059	0.238	1.268	0.973–1.654	0.184	1.203	0.934–1.548
Weekend	0.690*	1.995	1.073–3.707	0.437*	1.548	1.067–2.245	0.171	1.186	0.848–1.659
*Parental education level*									
2) No formal/Primary/Secondary									
Weekday	− 0.349	0.705	0.449–1.107	− 0.371*	0.690	0.496–0.962	− 0.104	0.901	0.641–1.268
Weekend	− 0.314	0.731	0.411–1.300	− 0.531*	0.588	0.391–0.885	0.122	1.130	0.701–1.822
3) Vocational									
Weekday	− 0.149	0.862	0.621–1.197	− 0.183	0.833	0.666–1.042	0.049	1.050	0.824–1.339
Weekend	− 0.091	0.913	0.598–1.394	− 0.368*	0.692	0.518–0.925	− 0.027	0.973	0.715–1.324
5) Master’s degree or higher									
Weekday	0.036	1.037	0.758–1.419	0.008	1.008	0.823–1.235	0.025	1.026	0.841–1.251
Weekend	0.297	1.346	0.864–2.097	0.077	1.080	0.810–1.440	0.141	1.151	0.874–1.518

Reference categories: Child’s gender: boy; Household income level: 3) 40,000–69,999 (€/year); Parental education level: 4) Bachelor’s degree. *p-value < 0.05, **p-value < 0.001

Children from higher-income households were more likely to meet the sleep quality guideline on weekends in 2020, and children from lower-income homes were less likely to meet the guideline on weekdays in 2021 (Table 6). In 2020, parents with higher education levels had their children less likely to meet the sleep quality guideline than parents with bachelor’s degrees.

**Table 6:** Coefficients and odds ratios (OR) of having minimum of good quality of sleep

	2019			2020			2021		
	Estimate	OR	95% CI	Estimate	OR	95% CI	Estimate	OR	95% CI
*Household income level (€/year)*									
1) Less than 20,000									
Weekday	− 0.145	0.865	0.527–1.421	− 0.176	0.839	0.577–1.219	− 0.408*	0.665	0.463–0.957
Weekend	− 0.314	0.730	0.417–1.280	− 0.115	0.891	0.581–1.368	− 0.204	0.816	0.530–1.254
2) 20,000–39,999									
Weekday	− 0.193	0.825	0.555–1.225	− 0.191	0.826	0.616–1.108	− 0.181	0.834	0.630–1.104
Weekend	0.062	1.064	0.656–1.728	0.076	1.079	0.757–1.539	− 0.145	0.865	0.626–1.194
4) 70,000–99,999									
Weekday	0.059	1.061	0.747–1.508	0.091	1.095	0.861–1.392	− 0.038	0.962	0.762–1.215
Weekend	0.162	1.176	0.766–1.806	0.175	1.191	0.903–1.571	0.056	1.058	0.811–1.379
5) 100,000 or more									
Weekday	0.165	1.179	0.749–1.857	0.238	1.268	0.937–1.716	0.092	1.096	0.832–1.443
Weekend	0.481	1.618	0.904–2.896	0.483*	1.621	1.138–2.310	0.169	1.184	0.862–1.626
*Parental education level*									
2) No formal /Primary/Secondary									
Weekday	0.025	1.025	0.601–1.750	0.228	1.256	0.821–1.921	− 0.013	0.987	0.684–1.424
Weekend	0.320	1.378	0.717–2.648	− 0.013	0.987	0.606–1.607	0.066	1.068	0.691–1.651
3) Vocational									
Weekday	0.165	1.179	0.820–1.697	0.101	1.106	0.847–1.445	0.062	1.064	0.827–1.370
Weekend	0.320	1.377	0.887–2.137	0.038	1.039	0.757–1.426	0.135	1.145	0.854–1.534
5) Master’s degree or higher									
Weekday	− 0.149	0.862	0.620–1.198	− 0.296*	0.744	0.596–0.929	0.061	1.063	0.858–1.318
Weekend	− 0.051	0.950	0.639–1.413	− 0.427*	0.653	0.506–0.842	− 0.048	0.953	0.745–1.218

Reference categories: Child’s gender: boy; Household income level: 3) 40,000–69,999 (€/year); Parental education level: 4) Bachelor’s degree. *p-value < 0.05, **p-value < 0.001

## Discussion

This study aimed to examine the prevalence of 2–6-year-old children in Finland meeting the WHO 24-h guidelines for ST, PA and sleep before and during the COVID-19 pandemic and its association with family SES, namely annual household income and parental level of education.

### Adherence to the WHO 24-h guidelines

Children met the ST guideline more often during weekdays than on weekends and the PA and sleep guidelines more often during weekends compared with weekdays at all timepoints. This may be caused by the different amounts of time available after work and pre-school hours in families on regular weekdays compared with weekend days or days off. Finnish pre-school days are known to be well structured [11] with physically active hours from around 10 am to 11 am and 3 pm to 4 pm [52], and it has been shown that during a pre-school day 97 percent of children engage in at least 1 h on outdoor PA [53]. This may further explain the prevalence of outdoor PA observed in this study, where the examined time was outside working and pre-school hours.

The adherence to the ST guideline was significantly lower on weekdays and weekends in 2020 during the restrictions due to the COVID-19 pandemic than the time before the pandemic outbreak in 2019. When the pandemic was still ongoing in 2021 but with fewer restrictions than in 2020, the adherence to the ST guideline nearly reached the level of 2019 on weekdays, but the weekend-ST remained the same as in 2020.

The prevalence of meeting the PA guideline was highest in 2020 during a period of relatively strict restrictions when attending social activities outside the home was curtailed. Other findings suggest decreased PA during the COVID-19 pandemic [37–40]. Some plausible explanations for these findings are noteworthy. For example, home yard features are known to be associated with young children’s outdoor play and PA [57, 58], also during the COVID-19 pandemic restrictions [42]. The outdoor environment is accessible to nearly everyone in Finland, so even though structured sports, for instance, were partly restricted, children could still freely engage in outdoor play.

The prevalence of meeting the sleep guidelines was relatively high compared to other findings [14, 18, 21] and similar to others [12, 15, 17], which can partly be explained by the differences in the measurement methods. A significant decline in sleep quality in 2021 compared with the two previous years is concerning, and continued monitoring of these results is needed to ascertain whether these observations become a longer-term trend or if sleep quality recovers to levels that were present before the pandemic.

### Associations with family socioeconomic status

Higher annual household income and higher parental education levels increased the odds of children meeting the ST guideline. These findings parallel the results for sleep duration, with lower household income and lower parental education levels decreasing the odds of meeting the sleep duration guideline. However, none of these results was shown to be significant throughout weekdays and weekend days at different timepoints. Other studies have indicated that higher parental education may mitigate young children’s ST [1, 27, 28], which is also connected to sleep [3, 4, 33]. However, sleep quality in the present research showed a contrary result in 2020, when children with parents with the highest education levels had their sleep quality less likely to meet the recommended levels. The disparity in this finding may reflect an imbalance between parental alertness towards the sleep guidelines and their child’s actual sleep, especially during the COVID-19 pandemic. Altogether, when discerning the significance of the overall results for both sleep guidelines, it appears that there is more favourable sleep among children from higher SES families.

In PA, a weekday-pattern by parental education level was detected, with children of less educated parents being more likely to meet the PA guideline than children of higher educated parents in all timepoints. Meeting the PA guideline by household income level was rather inconsistent; for instance, there was a lower likelihood of children meeting the PA guideline at both lower and higher income levels than at the mid-income level. Additionally, there was a contradiction in the highest income class, in which the likelihood of meeting the guideline was higher on weekends in 2020 but lower on weekdays in 2021. The reason for this finding is not yet apparent. Nonetheless, children from low- and middle-income countries are reported to meet the PA guideline more often than those from high-income countries [42]. Higher SES parents tend to work beyond regular working hours [59], and these parents may not have a clear division between work and free time compared with parents of lower income and education levels, especially during the COVID-19-pandemic. Less educated parents tended to work in sectors where remote work might not have been possible, with a clear distinction upheld between work and free time within families. These explanations are plausible but need to be confirmed in future research. Overall, household income level was not a significant factor in explaining children’s outdoor physical play in 2019, 2020 and 2021 in Finland.

### Further research and practical implications

In relation to the WHO guidelines for ST, PA and sleep in young children, in the time before and during the COVID-19 pandemic, ST is identified as one that has exceeded the guidelines the most. Overindulgence in daily screen viewing and video game playing are risk factors for night-time sleep, while quiet learning sedentary behaviour does not correlate with sleep duration in young children [6]. Further research should aim to more closely distinguish different aspects of ST, such as sedentary and non-sedentary ST, the extent to which children engage in them, and how these engagements affect the holistic well-being of children. For parents, this is very important, as the WHO Guidelines for physical activity and sedentary behaviour covering children five years and older [10], do not set a maximum daily time limit on ST engagement. The guidelines only recommend limiting recreational ST and having 60 min of moderate-to-vigorous PA and adequate good-quality sleep.

Further research is recommended to examine the bi-directional impact that ST, PA and sleep, coupled with the complexities of familial dyads, have on each other and on the well-being of young children. Lifestyle behaviours in adults and young children have been disrupted and altered significantly and in complex ways by the COVID-19 pandemic and may take some time before it becomes stabilized as the pandemic becomes endemic. Nonetheless, professionals working with young children and their families should acknowledge these changes and take practical steps to safeguard the good health and well-being of children and caregivers.

### Strengths and limitations

This study was implemented with a randomized nationally representative sample, using a valid and reliable online survey (SMALLQ®). To our knowledge, this is the first study to investigate in a national sample of children’s parent-reported digital media habits, outdoor physical play, sleep duration and sleep quality in Finland before and during the COVID-19 pandemic in 2019, 2020 and 2021. Lifestyle behaviour surveys may be affected by recall imprecision [60] and social desirability bias [61], and these were, respectively, minimized in the SMALLQ® by having the recall of ST, PA and sleep habits limited to a seven-day recall and by having the survey remain anonymous with no personal identifiable information.

Online surveys of this nature appear to have less of a reach to people from a lower SES [62], however, this sample achieved adequate national representativeness [51, 63]. This may be because the public early childhood education centres are feasible platforms to reach nearly all SES groups.

Moreover, it should be noted that the definition for meeting the WHO guidelines differs across studies and therefore comparisons of the results should be done with caution. In this study, the national guidelines for under school-aged children [11] were acknowledged for the cultural context of the sample. These applications concerned the importance of outdoor play in children’s PA and the limitation of continuous sedentary time to 1 h.

## Conclusion

Adherence to the ST guideline among 2–6-year-old children was lowest and to the PA guideline highest during the most stringent COVID-19 restrictions in 2020. Sleep quality was reduced during the second year of the pandemic in 2021. High family SES may increase the odds of children meeting the ST and sleep guidelines, but engaging in outdoor PA is more complex by family SES. Professionals working with young children and their families should continue to monitor and respond to unfavourable changes in children’s ST, PA and sleep during and beyond the COVID-19 pandemic to safeguard the future health of children. More research is needed to track the causality of these relationships.

## Supplementary Information

**Additional file 1.** Coefficients and odds ratios (OR) of child meeting the WHO 24-hour guidelines in 2019, 2020, and 2021.

## Data Availability

The datasets used and analysed during the current study are available from the corresponding author upon reasonable request.

## Abbreviations

COVID-19: Coronavirus disease 2019
PA: Physical activity
SES: Socioeconomic status
ST: Screen time
WHO: World Health Organization
