# Supplementary material for: Meeting the WHO 24-h guidelines among 2–6-year-old children by family socioeconomic status before and during the COVID-19 pandemic: a repeated cross-sectional study
Source: J Act Sedentary Sleep Behav. 2023 Jan 3;2:2. doi: 10.1186/s44167-022-00010-4 (PMC9807983; doi:10.1186/s44167-022-00010-4)
Supplement: Supplementary file 1 — Additional file 1. Coefficients and odds ratios (OR) of child meeting the WHO 24-hour guidelines in 2019, 2020, and 2021. [file 44167_2022_10_MOESM1_ESM.pdf]

Additional file 1

Coefficients and odds ratios (OR) of child meeting the WHO 24-hour guidelines in 2019, 2020, and 2021.

	2019 Weekday			2019 Weekend			2020 Weekday			2020 Weekend			2021 Weekday			2021 Weekend		
	Estimate	OR	95% CI	Estimate	OR	95% CI	Estimate	OR	95% CI	Estimate	OR	95% CI	Estimate	OR	95% CI	Estimate	OR	95% CI
**ST[1]**																		
Child's age	**-0.153***	0.858	0.776–0.949	**-0.435****	0.648	0.571–0.734	**-0.263****	0.769	0.715–0.827	**-0.475****	0.622	0.563–0.687	**-0.194****	0.823	0.766–0.886	**-0.460****	0.631	0.571–0.699
Child's gender	-0.156	0.856	0.703–1.041	0.014	1.014	0.795–1.295	-0.026	0.974	0.847–1.120	0.014	1.014	0.847–1.214	-0.086	0.917	0.797–1.056	-0.196	0.822	0.681–0.992
Parent's age	0.036	1.036	0.928–1.157	0.060	1.062	0.926–1.218	-0.032	0.968	0.897–1.045	-0.032	0.968	0.878–1.067	**-0.097***	0.907	0.839–0.981	**-0.140***	0.870	0.781–0.968
Parent's gender	0.055	1.057	0.800–1.395	0.122	1.130	0.799–1.597	0.001	1.001	0.816–1.229	0.111	1.117	0.864–1.443	-0.167	0.846	0.694–1.032	-0.012	0.988	0.756–1.290
**Outdoor PA[2]**																		
Child's age	-0.060	0.942	0.851–1.042	-0.063	0.939	0.816–1.080	0.008	1.008	0.941–1.081	0.012	1.012	0.893–1.147	-0.047	0.954	0.889–1.023	-0.002	0.998	0.889–1.121
Child's gender	**-0.314***	0.731	0.602–0.888	**-0.321***	0.726	0.554–0.950	**-0.307****	0.736	0.642–0.842	**-0.511****	0.600	0.471–0.764	-0.215*	0.807	0.703–0.925	**-0.311***	0.733	0.588–0.913
Parent's age	**-0.145***	0.865	0.773–0.968	0.044	1.045	0.898–1.217	-0.044	0.957	0.887–1.031	0.010	1.011	0.888–1.151	-0.037	0.964	0.895–1.038	0.059	1.061	0.943–1.195
Parent's gender	0.011	1.011	0.764–1.336	-0.004	0.996	0.679–1.461	0.033	1.034	0.848–1.260	-0.113	0.893	0.631–1.263	0.096	1.101	0.911–1.331	-0.081	0.922	0.688–1.236
**Sleep Duration[3]**																		
Child's age	**0.586****	1.796	1.587–2.033	**0.289****	1.335	1.143–1.558	**0.442****	1.556	1.431–1.692	**0.183***	1.201	1.079–1.337	**0.418***	1.519	1.395–1.653	**0.204****	1.227	1.099–1.369
Child's gender	-0.040	0.961	0.752–1.228	-0.242	0.785	0.565–1.092	-0.033	0.967	0.822–1.138	-0.025	0.976	0.783–1.215	-0.058	0.944	0.800–1.114	0.155	1.168	0.935–1.459
Parent's age	**-0.269****	0.764	0.665–0.878	**-0.210***	0.811	0.690–0.952	**-0.129***	0.879	0.803–0.962	**-0.191***	0.826	0.739–0.923	**-0.144***	0.866	0.793–0.946	0.010	1.010	0.903–1.129
Parent's gender	-0.209	0.812	0.583–1.131	**-0.512***	0.599	0.395–0.911	**-0.387***	0.679	0.544–0.847	**-0.652****	0.521	0.394–0.688	**-0.311***	0.733	0.587–0.915	**-0.430***	0.650	0.490–0.863
**Sleep Quality[4]**																		
Child's age	**0.510****	1.665	1.443–1.920	**0.613****	1.846	1.549–2.201	**0.500****	1.649	1.496–1.819	**0.624****	1.867	1.663–2.095	**0.460****	1.584	1.438–1.744	**0.572****	1.772	1.581–1.986
Child's gender	0.125	1.133	0.865–1.485	0.302	1.353	0.969–1.889	**-0.191***	0.826	0.686–0.995	-0.066	0.936	0.753–1.164	-0.038	0.962	0.808–1.147	0.168	1.183	0.967–1.448
Parent's age	-0.010	0.990	0.851–1.152	0.032	1.033	0.851–1.253	-0.079	0.924	0.835–1.022	-0.084	0.920	0.820–1.031	-0.062	0.940	0.856–1.032	-0.050	0.951	0.859–1.054
Parent's gender	0.292	1.339	0.901–1.990	0.014	1.014	0.638–1.613	0.158	1.171	0.888–1.543	0.084	1.087	0.791–1.494	0.148	1.160	0.901–1.494	0.018	1.019	0.766–1.355

[1] maximum of 60 minutes of ST, [2] minimum of 60 minutes of outdoor PA, [3] minimum of 11/10/9 hours (2/3–5/6 years) of sleep, [4] minimum of good quality of sleep; * p-value <0.05, **p-value <0.001. When analysed missing data of outcome variables and other variables of the dataset, a variable of parental concerns on their child's digital media use appeared to be statistically significant predictor of missing values (data not shown). Missing values are supposed to be missing at random (MAR) when a variable of parental concern was added to the model as an auxiliary variable (55,56).
